# Draft Genome Sequences of Type Strains of Gordonibacter faecihominis, Paraeggerthella hongkongensis*, Parvibacter caecicola,* Slackia equolifaciens, Slackia faecicanis, and Slackia isoflavoniconvertens

**DOI:** 10.1128/MRA.01532-18

**Published:** 2019-01-10

**Authors:** Nicolas Danylec, Dominic A. Stoll, Andreas Dötsch, Melanie Huch

**Affiliations:** a Max Rubner-Institut, Federal Research Institute of Nutrition and Food, Department of Safety and Quality of Fruit and Vegetables, Karlsruhe, Germany; b Max Rubner-Institut, Federal Research Institute of Nutrition and Food, Department of Physiology and Biochemistry of Nutrition, Karlsruhe, Germany; Georgia Institute of Technology

## Abstract

Here, we report the annotated draft genome sequences of six type strains of the family *Eggerthellaceae*, Gordonibacter faecihominis JCM 16058, Paraeggerthella hongkongensis DSM 16106, Parvibacter caecicola DSM 22242, Slackia equolifaciens DSM 24851, Slackia faecicanis DSM 17537, and Slackia isoflavoniconvertens DSM 22006.

## ANNOUNCEMENT

*Eggerthellaceae* are typical members of the mammalian gut and have been isolated from healthy humans (1). However, Eggerthella sinensis and Paraeggerthella hongkongensis were also associated with bacteremia (2). Some members have been reported to metabolize secondary plant compounds, especially polyphenols. Slackia equolifaciens converts resveratrol into dihydroresveratrol (3), Gordonibacter urolithinfaciens metabolizes ellagic acid into urolithin derivatives (4), and Asaccharobacter celatus, S. equolifaciens, and Slackia isoflavoniconvertens show the ability to metabolize daidzein into (*S*)-equol (5–7).

We announce the first draft genome sequences of Parvibacter caecicola DSM 22242^T^, S. equolifaciens DSM 24851^T^, Slackia faecicanis DSM 17537^T^, and Gordonibacter faecihominis JCM 16058^T^. Although draft genome sequences of P. hongkongensis and S. isoflavoniconvertens strains were previously published (8), here we announce the sequences of the respective type strains DSM 16106 and DSM 22006.

Selected strains were obtained from the German Collection of Microorganisms and Cell Cultures (DSMZ) and from the Japanese Culture Collection (JCM). Strains were cultured under anaerobic conditions of N_2_-CO_2_ (80:20) in flushed brain heart infusion medium (Merk) supplemented with 0.5% yeast extract, 0.05% l-cysteine monohydrochloride (Roth), 1 mg ml^−1^ resazurin sodium salt, 2.5 mg l^−1^ heme solution, and 2 μg ml^−1^ vitamin K_1_ solution (Sigma-Aldrich). A blood and tissue kit (Qiagen) was used for DNA extraction. DNA was quantified with a dsDNA HS assay on a Qubit version 2.0 fluorometer (Thermo Fischer Scientific) according to the manufacturer’s instructions and adjusted to a concentration of 2.0 ng µL^−1^.

Preparation of sequencing libraries as well as data processing were done as previously described (9). In brief, sequencing libraries were built using a Nextera XT DNA library prep kit and a Nextera XT index kit (Illumina). A paired-end (2 × 300 bp) sequencing run was performed on an Illumina MiSeq instrument using a 600-cycle version 3 kit. Reads were quality trimmed using Trimmomatic version 0.36 (10) and were assembled using SPAdes version 3.11.1 with default settings in the careful mode (11, 12). Adequate trimming was checked by mapping the adapter sequences to the assembled contigs using Bowtie 2 version 2.3.3.1 (13). To exclude contamination, the contigs were aligned to the genome of coliphage PhiX174 (GenBank accession number NC_001422) using a BLASTn search (14). All contigs of <500 bp were excluded. To calculate genome coverage of each strain (Table 1), trimmed reads were mapped against remained contigs by using Bowtie 2. Draft genome sequences were annotated using the automated NCBI Prokaryotic Genome Annotation Pipeline (15). The assembly metrics and annotated features are given in Table 1.

**TABLE 1:** Accession numbers, assembly metrics, and annotated features of the sequenced *Eggerthellaceae* type strains

Species	Strain	GenBank accession no.	Average coverage (×)	No. of contigs	Genome assembly size (bp)	*N*_50_ value (bp)	G+C content (mol%)	No. of coding genes	No. of rRNAs	No. of tRNAs	No. of ncRNAs^*a*^
Gordonibacter faecihominis	JCM 16058	QIBV00000000	141.9	64	3,236,830	107,270	66.5	2,706	6	47	3
Paraeggerthella hongkongensis	DSM 16106	QICD00000000	160.2	67	2,801,926	91,131	60.9	2,365	9	50	3
Parvibacter caecicola	DSM 22242	QIBY00000000	157.9	64	2,479,984	114,979	62.5	1,950	7	46	3
Slackia equolifaciens	DSM 24851	QIBX00000000	168.1	51	2,745,526	109,974	59.8	2,149	10	47	3
Slackia faecicanis	DSM 17537	QICB00000000	246.4	20	1,995,252	520,619	63.0	1,648	7	58	3
Slackia isoflavoniconvertens	DSM 22006	QIBZ00000000	224.1	53	2,253,648	162,276	57.7	1,844	9	61	3

a ncRNAs, noncoding RNAs.

ResFinder 3.0 (16) detected the tetracycline resistance determinant *tet*(W) in S. equolifaciens DSM 24851^T^, S. faecicanis DSM 17537^T^, and S. isoflavoniconvertens DSM 22006^T^.

According to *in silico* DNA-DNA hybridization analyses (17), G. faecihominis JCM 16058^T^ and G. urolithinfaciens DSM 27213^T^ are closely related, with a similarity value of 88.7%, and therefore may represent the same species. However, this should be validated with a polyphasic approach.

### Data availability.

The draft genome sequences of these *Eggerthellaceae* type strains have been deposited at DDBJ/ENA/GenBank under the BioProject number PRJNA473639, and the described accession numbers in this publication are listed in Table 1. For all sequences, the first versions of the accession numbers are described in this paper. Additionally, this BioProject includes three draft genomes of G. urolithinfaciens DSM 27213 (QIBW00000000), E. sinensis DSM 16107 (QICC00000000), and A. celatus DSM 18785 (QICA00000000).
